# Influence of Vertical Trabeculae on the Compressive Strength of the Human Vertebra

**DOI:** 10.1002/jbmr.207

**Published:** 2010-08-16

**Authors:** Aaron J Fields, Gideon L Lee, X Sherry Liu, Michael G Jekir, X Edward Guo, Tony M Keaveny

**Affiliations:** 1 Orthopaedic Biomechanics Laboratory, Department of Mechanical Engineering, University of California Berkeley, CA, USA; 2 Bone Bioengineering Laboratory, Department of Biomedical Engineering, Columbia University New York, NY, USA; 3 Department of Bioengineering, University of California Berkeley, CA, USA

**Keywords:** BONE STRENGTH, SPINE, BIOMECHANICS, FINITE-ELEMENT ANALYSIS, OSTEOPOROSIS

## Abstract

Vertebral strength, a key etiologic factor of osteoporotic fracture, may be affected by the relative amount of vertically oriented trabeculae. To better understand this issue, we performed experimental compression testing, high-resolution micro–computed tomography (µCT), and micro–finite-element analysis on 16 elderly human thoracic ninth (T_9_) whole vertebral bodies (ages 77.5 ± 10.1 years). Individual trabeculae segmentation of the µCT images was used to classify the trabeculae by their orientation. We found that the bone volume fraction (BV/TV) of just the vertical trabeculae accounted for substantially more of the observed variation in measured vertebral strength than did the bone volume fraction of all trabeculae (*r*^2^ = 0.83 versus 0.59, *p* < .005). The bone volume fraction of the oblique or horizontal trabeculae was not associated with vertebral strength. Finite-element analysis indicated that removal of the cortical shell did not appreciably alter these trends; it also revealed that the major load paths occur through parallel columns of vertically oriented bone. Taken together, these findings suggest that variation in vertebral strength across individuals is due primarily to variations in the bone volume fraction of vertical trabeculae. The vertical tissue fraction, a new bone quality parameter that we introduced to reflect these findings, was both a significant predictor of vertebral strength alone (*r*^2^ = 0.81) and after accounting for variations in total bone volume fraction in multiple regression (total *R*^2^ = 0.93). We conclude that the vertical tissue fraction is a potentially powerful microarchitectural determinant of vertebral strength. © 2011 American Society for Bone and Mineral Research.

## Introduction

Osteoporosis decreases vertebral strength owing to loss of bone mass and deterioration of bone microarchitecture. Osteoporosis also increases the anisotropy of the trabecular structure(1,2) because more horizontal trabecular bone is lost than vertical trabecular bone.(3) The relative role of vertical versus horizontal trabecular bone on vertebral strength remains poorly understood and may provide new insight into the etiology of age- and disease-related vertebral fractures and ultimately could lead to improved prediction of vertebral strength and assessment of fracture risk. Previous work on isolated specimens of trabecular bone found that the bone volume fraction of vertical trabeculae better predicted overall mechanical behavior than did the bone volume fraction (BV/TV) of the entire specimen(4); vertical trabeculae also failed in the greatest number.(5) However, extrapolation of these findings to the whole vertebral body is not obvious because the biomechanical behavior of the whole vertebra has a substantial and complex contribution from the cortical shell,(6–11) which could alter the effect of vertical trabeculae. Based on our previous findings that the roles of the cortical shell and trabecular microarchitecture—such as bone volume fraction—may be largely independent,(12) we hypothesized that vertebral strength is better explained by the bone volume fraction of the vertical trabeculae than by the bone volume fraction of all trabeculae and that the cortical shell does not alter the effect of vertical trabeculae on the biomechanical behavior of the vertebra.

## Materials and Methods

### Specimen preparation and micro–computed tomographic (µCT) scanning

Sixteen whole thoracic ninth (T_9_) vertebrae were obtained fresh frozen from human cadaver spines (age 77.5 ± 10.1 years, 53 to 97 years, *n* = 10 male, *n* = 6 female) with no history of metabolic bone disorders. As described elsewhere in more detail,(12) the posterior elements were removed, and each isolated vertebral body was µCT scanned with a 30-µm voxel size (Scanco 80, Scanco Medical AG; Brüttisellen, Switzerland). The scans were coarsened to 60-µm voxel size, and the hard tissue and marrow were segmented using a global threshold value (Scanco). The bone tissue in the trabecular compartment then was digitally isolated from the cortical shell and endplates using a custom script (IDL 6.2, ITT Visualization Information Solutions, Boulder, CO, USA), described in detail elsewhere.(7,13) Briefly, the script uses a moving average of the thickness of the cortical shell and of the endplates to account for the thin and porous nature of these structures and to determine the boundary between these structures and any adjacent trabeculae.

### Orientation-related morphology parameters

Morphologic analyses were performed to classify the orientation of trabeculae in the trabecular compartment. Individual trabeculae were identified using the individual trabeculae segmentation (ITS) technique(4) and classified by orientation with respect to the superoinferior anatomic axis: vertical (0 to 30 degrees), oblique (31 to 60 degrees), or horizontal (61 to 90 degrees). We evaluated the following orientation-related morphologic parameters for the trabecular compartment: bone volume fraction (BV/TV); bone volume fraction of vertical trabeculae (vBV/TV); bone volume fraction of oblique trabeculae (oBV/TV); bone volume fraction of horizontal trabeculae (hBV/TV); vertical tissue fraction (vBV/BV), the volume of vertical trabeculae divided by the volume of all trabeculae; oblique tissue fraction (oBV/BV), the volume of oblique trabeculae divided by the volume of all trabeculae; and horizontal tissue fraction (hBV/BV), the volume of horizontal trabeculae divided by volume of all trabeculae. We also evaluated two variants of the vertical tissue fraction: vBV/BV_vertebra_, the volume of vertical trabeculae divided by the total volume of bone tissue in the vertebral body, that is, trabecular bone + cortical shell + endplates; and vBV_vertebra_/BV_vertebra_, the volume of vertical bone tissue in the vertebral body, that is, vertical trabeculae + cortical shell, divided by the total volume of bone tissue in the vertebral body.

### Biomechanical testing

To characterize the biomechanical properties of the vertebral bodies, destructive compression testing was performed after µCT scanning. Details of the biomechanical tests are described elsewhere in more detail.(12,14,15) Briefly, these experiments were conducted using a screw-driven load frame with a lockable ball joint to allow the top platen of the load frame to rest flat on the vertebrae during compression. The vertebrae were first placed between polymethyl methacrylate (PMMA) endcaps to ensure planoparallel ends.(16,17) The compression tests were performed in displacement control at a slow strain rate (∼0.05% to 0.5% strain/second) after cyclic preconditioning.(12) Vertebral strength *F*_ult_ was defined as the peak force achieved during the loading cycle.

### Finite-element (FE) modeling

To identify the load-bearing tissues and to examine the interaction between the cortical shell and the trabeculae in each orientation, we performed high-resolution finite-element analysis. Two finite-element models—one model of each intact vertebra and one model of each vertebra with the cortical shell virtually removed—were created from the coarsened µCT scans.(12,13) Each 60-µm cubic voxel in the scans was converted into an eight-noded brick element to create a finite-element model of the entire vertebral body. Element size was chosen based on a numerical convergence study.(13) Linear finite-element analysis was conducted for each model to 1% apparent compressive strain via simulated layers of PMMA (elastic modulus 2.5 GPa and Poisson's ratio of 0.3(18)) extended from the inferior and superior endplates. All bone elements were assigned the same homogeneous and isotropic hard tissue material properties: elastic modulus 10 GPa,(19) Poisson's ratio of 0.3. To determine the effect of the cortical shell, a second finite-element model for each vertebra with the cortical shell removed was analyzed while keeping all other model inputs unchanged. Models contained 25 to 80 million elements. A highly scalable, implicit parallel finite-element framework (Olympus(20)) was used for all analyses. These analyses were performed on an IBM Power4 supercomputer (Datastar, San Diego Supercomputer Center, San Diego, CA, USA) and required up to 880 processors in parallel and 1800 GB of memory.

A number of outcomes from the finite-element analyses were used to characterize the biomechanical behavior of the vertebral bodies. Stiffness of the intact vertebra *K*_intact_ and of the trabecular compartment *K*_trab_ were defined as the ratio of the reaction force to the applied displacement in the models with and without the cortical shell, respectively. Stress distributions in the models were used to identify the major load-bearing tissues in the vertebrae. These load-bearing tissues were defined as the elements having von Mises stress above the 75th percentile in each model.(21) Varying the cutoff von Mises stress between the 75th and 90th percentiles did not alter our conclusions.

### Statistics

The independent effects of the orientation-related morphology parameters on measured vertebral strength and finite-element-predicted vertebral stiffness were assessed with the Pearson correlation coefficient. To quantify the interaction between the cortical shell and the trabeculae in each orientation, relationships between stiffness and bone volume fraction were determined with intact stiffness and trabecular stiffness as the outcome. The statistically significant relationships then were compared using paired *t* tests on the regression slopes and on the predicted residuals. The percentage of load-bearing tissue was compared across orientations using paired *t* tests with Bonferroni adjustments for multiple comparisons. Multiple linear regression analysis also was used to investigate the combined roles of bone volume fraction and vertical tissue fraction in vertebral strength. All statistical tests (JMP 7.0, SAS Institute, Cary, NC, USA) were taken as significant at *p* < .05.

## Results

Over half the trabecular tissue was vertically oriented, more than twice the proportion of trabecular tissue that was either obliquely or horizontally oriented (Table 1). Given the highly porous nature of the cohort (BV/TV = 14% ± 3%, mean ± SD), the bone volume fraction of vertical trabeculae (vBV/TV) ranged from just 4% to 11%.

**Table 1:** Orientation-Related Morphology Parameters for *n* = 16 Human T_9_ Vertebral Bodies

	Mean	SD	CV (%)	Range
Trabecular bone volume fraction				
Total, BV/TV (%)	13.5	3.3	24.4	7.8–18.7
Vertical, vBV/TV (%)	7.2	2.2	30.6	3.9–11.4
Oblique, oBV/TV (%)	3.1	0.7	22.6	1.9–4.5
Horizontal, hBV/TV (%)	3.2	0.8	25.0	1.9–4.5
Trabecular tissue fraction				
Vertical, vBV/BV (%)	52.7	5.2	9.9	45.0–64.3
Oblique, oBV/BV (%)	22.2	2.3	10.4	14.5–28.3
Horizontal, hBV/BV (%)	24.1	3.8	15.8	19.2–26.5

**Fig. 1:**
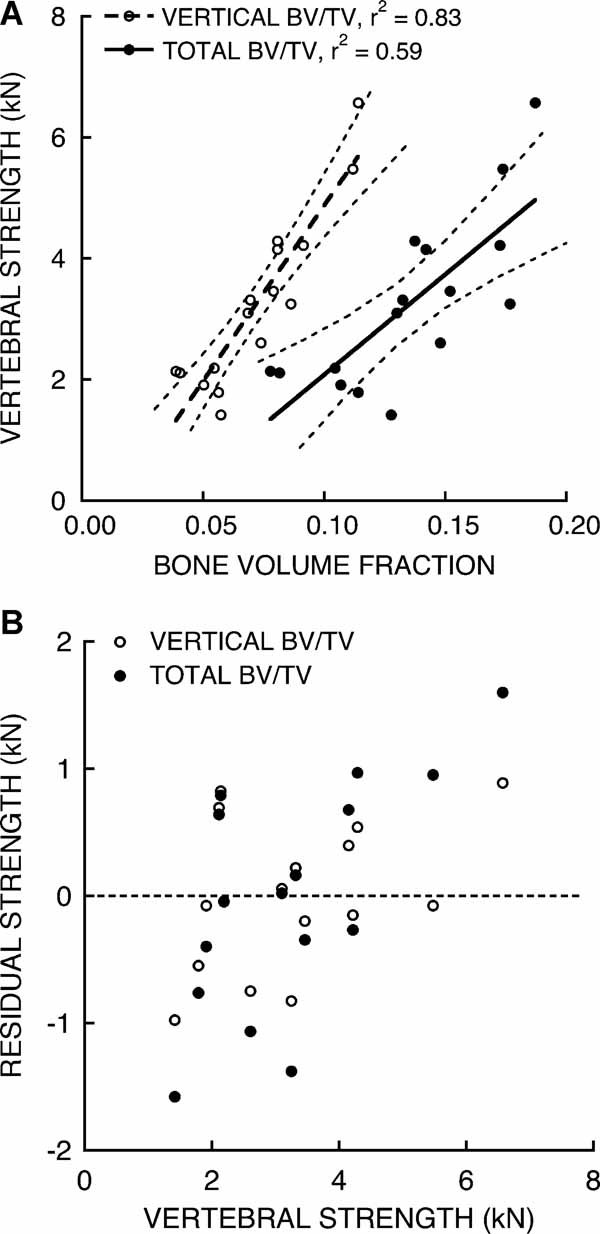
Variations in measured vertebral strength were predicted better by variations in the bone volume fraction (BV/TV) of vertical trabeculae than by variations in the BV/TV of all trabeculae. (*A*) Strength–BV/TV regressions for total BV/TV and vertical BV/TV. Dashed lines show the 95% confidence bands for each fitted line. (*B*) Residuals from predicted strength using the BV/TV of vertical trabeculae as the predictor (absolute residual = 0.5 ± 0.3 kN) were 20% lower, on average (*p* < .005, paired *t* test), than the residuals from predicted strength using the BV/TV of all trabeculae as the predictor (0.7 ± 0.5 kN).

The variation in both experiment-measured vertebral strength and finite-element-predicted vertebral stiffness was most associated with the bone volume fraction of vertical trabeculae (Table 2). Compared with the bone volume fraction of all trabeculae, the bone volume fraction of vertical trabeculae accounted for substantially more of the variation in vertebral strength (*r*^2^ = 0.83 versus *r*^2^ = 0.59; Fig. 1*A*) and had significantly lower residuals (*p* < .005, paired *t* test on residuals; Fig. 1*B*). The bone volume fractions of oblique and horizontal trabeculae were not associated with vertebral strength and were weakly associated with vertebral stiffness. As expected, the bone volume fraction of vertical, oblique, and horizontal trabeculae were each correlated with total bone volume fraction (*r*^2^ = 0.90, 0.80, and 0.51, respectively).

**Table 2:** Independent Effect (Pearson's Correlation Coefficient *r*) of the Orientation-Related Morphology Parameters on Measured Vertebral Strength (*F*_ult_), Intact Vertebral Stiffness (*K*_intact_), and Trabecular Stiffness (*K*_trab_) for *n* = 16 Vertebral Bodies

	*F*_ult_	*K*_intact_	*K*_trab_
Trabecular bone volume fraction			
Total, BV/TV	0.77^c^	0.93^c^	0.90^c^
Vertical, vBV/TV	0.91^c^	0.97^c^	0.95^c^
Oblique, oBV/TV	NS	0.72^b^	0.68^b^
Horizontal, hBV/TV	NS	0.53^a^	NS
Trabecular bone tissue fraction			
Vertical, vBV/BV	0.90^c^	0.71^b^	0.75^c^
Oblique, oBV/BV	−0.55^a^	NS	NS
Horizontal, hBV/BV	−0.76^c^	−0.58^a^	−0.62^b^

NS = not significant.

a *p* < .05.

b *p* < .01.

c *p* < .001.

**Fig. 2:**
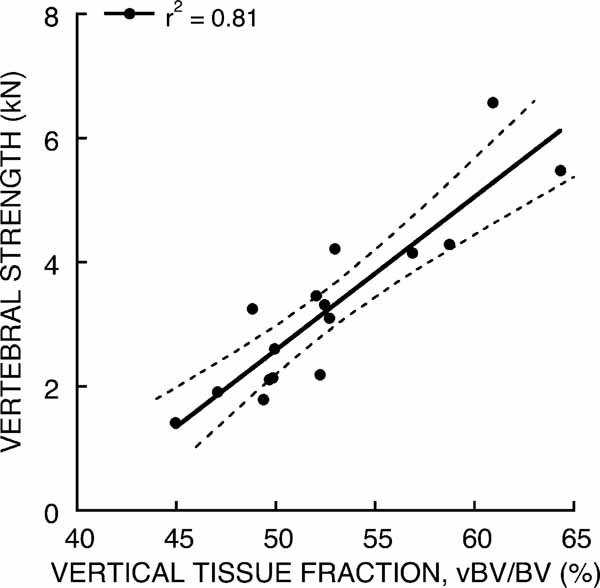
Variations in measured vertebral strength were associated with variations in vertical tissue fraction—the bone volume of vertical trabeculae divided by the bone volume of all trabeculae (*p* < .001).

After accounting for the variation in total bone volume fraction (BV/TV), the vertical trabeculae remained most strongly associated with vertebral strength by way of variations in vertical tissue fraction (vBV/BV: *r*^2^ = 0.81; Table 2 and Fig. 2). Expressing the vertical trabeculae as a fraction of all the bone tissue in the vertebral body worsened the correlation (vBV/BV_vertebra_: *r*^2^ = 0.56, *p* < .001), as did including the cortical shell in the measure of vertically oriented bone tissue (vBV_vertebra_/BV_vertebra_: *r*^2^ = 0.17, *p* = .12). The vertical tissue fraction (vBV/BV) was only weakly correlated with total bone volume fraction (BV/TV; *r*^2^ = 0.28, *p* = .04). In a multiple linear regression model with vertebral strength as the outcome, both the vertical tissue fraction (vBV/BV, *p* < .0001) and the total bone volume fraction (BV/TV, *p* < .0005) were significant predictors (BV/TV alone; *r*^2^ = 0.59; BV/TV and vBV/BV: *R*^2^ = 0.93).

**Fig. 3:**
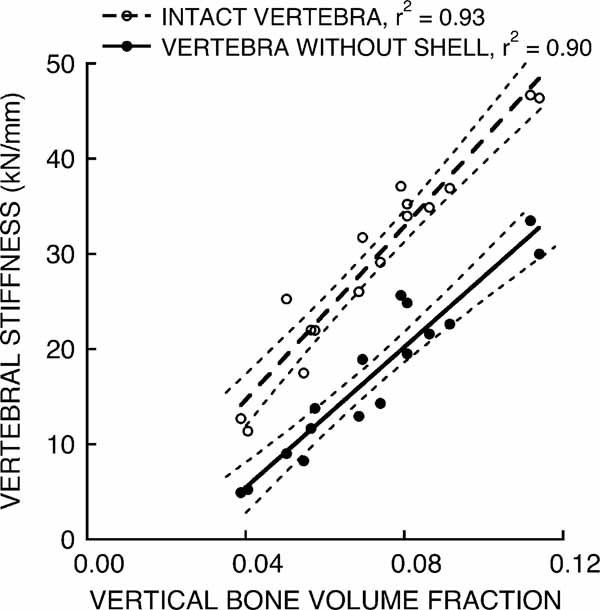
Variations in finite-element-predicted vertebral stiffness for the intact vertebra and for the trabecular compartment were associated with variations in the bone volume fraction of vertical trabeculae.

Results from the finite-element models revealed that the physical presence of the cortical shell did not appreciably alter the degree of association between the bone volume fraction of vertical trabeculae and vertebral stiffness (Fig. 3). Without the shell, the vertebral bodies were less stiff (downward shift in the regression data), but the interaction between the cortical shell and the bone volume fraction of vertical trabeculae varied little across individuals (no difference in residuals: *p* = .92; similar regression slopes: *p* = .07). Similarly, removing the shell had no significant effect on the relationship between the bone volume fraction of oblique trabeculae and vertebral stiffness (no difference in residuals: *p* = .23; no difference in regression slopes: *p* = .50). The bone volume fraction of horizontal trabeculae was not significantly associated with the stiffness of the vertebra without the shell.

**Fig. 4:**
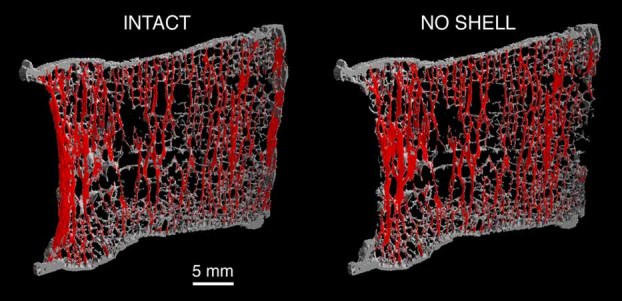
Midsagittal section (*left*) from a human T_9_ vertebra showing the typical load paths—the bone tissue with von Mises stress in the highest quartile, *red*—predicted by finite-element analysis. In this vertebra, approximately 48% of the load paths belonged to the vertical trabecular bone. Removing the cortical shell (*right*) did not alter the vertical nature of the load paths.

The stress distributions from the finite-element models revealed that the major load paths in the vertebrae were vertically oriented (Fig. 4). Of the tissue that was stressed in the 75th percentile, 41.2% ± 6.3% was composed of the vertical trabecular bone and 27.0% ± 5.6% was composed of the cortical shell. By comparison, significantly less of the tissue stressed in the 75th percentile resided in the oblique (10.4% ± 1.8%, *p* < .0001) and horizontal trabeculae (8.6% ± 2.2%, *p* < .0001). Removing the cortical shell did not alter the vertical nature of the load paths (Fig. 4); as expected, it mainly resulted in unloading of the peripheral trabeculae.(7)

## Discussion

These results confirmed our hypothesis, demonstrating that variation in vertebral strength across individuals was primarily due to variations in the bone volume fraction of vertical trabeculae. This is so because the major load paths in the vertebrae were parallel columns of vertically oriented bone—the vertical trabeculae and the cortical shell. Whereas variations in the amount of vertical trabeculae had an important role in vertebral strength, variations in the amount of cortical tissue had a minor role.(12) Moreover, the cortical shell did not alter the association between the bone volume fraction of vertical trabeculae and vertebral stiffness. As with many microarchitecture parameters,(12,22) the bone volume fraction of vertical trabeculae was highly associated with total bone volume fraction. To remove any influence of variations in total bone volume fraction, we introduced a new parameter—vertical tissue fraction (vBV/BV). Most interestingly, this new parameter was only weakly associated with total bone volume fraction, and yet it retained its high correlation with vertebral strength. Further, both the vertical tissue fraction and total bone volume fraction remained highly significant in a multiple linear regression model to predict vertebral strength. As such, vertical tissue fraction represents a new indicator of bone quality.(23,24) While requiring confirmation in larger studies, these collective findings demonstrate a new and potentially powerful microarchitectural determinant of vertebral strength.

Our earlier work on isolated specimens of trabecular bone showed a strong association between vertical trabeculae and biomechanical behavior(4,5)—these new results extend those previous findings to whole vertebrae. In addition to orientation, the structure of individual trabeculae, for example, plate versus rod, also may have an important effect on biomechanical behavior.(4,25,26). Liu and colleagues predicted that more vertical plates fail than vertical rods during axial compression of vertebral trabecular bone.(5) Since the effect of vertical trabeculae reported here includes both plates and rods, it is possible that considering the number of vertical plates may further improve predictions of vertebral strength. This remains a topic of ongoing research and may require analyzing images with a higher spatial resolution to accurately characterize the rodlike trabeculae.(27) The excellent agreement between vBV/TV derived from images with a 25-µm voxel size compared with a coarsened 60-µm voxel size (*r*^2^ = 0.99 and slope = 0.94 for *n* = 19 samples of tibial trabecular bone; data not shown) suggests that analyzing images with a higher spatial resolution is unlikely to change our conclusions regarding the effect of vBV/TV on vertebral strength.

These findings have potentially important clinical implications for microarchitecture analysis of bone strength. Compared with the role of the traditional microarchitecture parameters (Tb.Th*, Tb.Sp*, Tb.N*, SMI, and DA) that we evaluated previously for this same cohort(12) and which have been evaluated by others,(22,28) the vertical tissue fraction parameter vBV/BV was more highly associated with vertebral strength and stiffness. In fact, vBV/BV was as good a predictor of vertebral strength as the finite-element models (*r*^2^ = 0.76, *K*_intact_ versus *F*_ult_)—although this may be specific to the compressive loading conditions. Thus this new parameter may represent an aspect of microarchitecture with the most significance from a biomechanical perspective. Of those same microarchitecture parameters assessed previously,(12) only SMI was associated with vBV/BV (*r*^2^ = 0.64, *p* < .001). Previous studies have shown that trabecular microarchitecture assessed in the spine(29,30) and at peripheral sites(31–33) is associated with osteoporotic fracture in the spine. It remains to be seen if this new microarchitecture parameter, whether measured in the spine or at peripheral sites, can improve fracture risk assessment.

Another issue related to the importance of trabecular microarchitecture is the relative role of vertical versus horizontal trabeculae. It is thought that horizontal trabeculae act as stabilizing cross-braces to the vertical trabeculae that undergo bending and buckling.(5,34,35) However, across individuals, we found that variations in the relative number of horizontal trabeculae were not associated with variations in vertebral compressive strength. Thus, despite their theoretical importance, variations in the number of horizontal trabeculae across individuals appear to be much less important than variations in the number of vertical trabeculae in terms of accounting for observed variations in vertebral strength. We did not address intravertebral variations in thickness or spacing of either the vertical or the horizontal trabeculae.(3,36–38) It is unclear whether considering such variations can further improve assessment of vertebral strength.

A notable feature of this study design was our combined experimental and computational approach, which allowed us to explain the mechanisms underlying the high statistical correlation observed between the number of vertical trabeculae and vertebral strength. The repeated-measures analysis of the finite-element models with versus without the thin cortical shell provided a statistically powerful and unique means of understanding the contribution of the shell to this aspect of whole-vertebral biomechanical behavior. Regarding external validity, the consistency of our findings across a cohort with a wide range of biomechanical properties and morphologies suggests that our findings should apply quite generally, although confirmation in larger and younger cohorts is required. For example, we found that there was only a small effect of variations in the cortical shell, which may have been due to the small variation in cortical mass fraction observed across individuals (mean ± SD = 14.6% ± 3.7%). It is possible that a larger cohort with younger individuals may have greater variations in the cortical shell, which may increase its role.

We focused on compressive loading because functional loads in the spine are primarily compressive in nature.(39) For compression, the stresses in the vertebra are vertically oriented. Since many osteoporotic vertebral fractures are wedge fractures,(40) the response to forward flexion may have additional clinical relevance. Forward flexion is not well understood in terms of how the extra bending moment is distributed between the spinal musculature and the vertebral body.(39) If some of the bending moment is taken up directly by the vertebral body, we still would expect the major load paths to remain vertically oriented because the bending moment would not introduce any multiaxial loads but instead would produce a nonuniform distribution of vertically oriented stress. This nonuniform distribution likely would result in higher stresses anteriorly.(41,42) In this case, it is possible that measures of vertical tissue fraction in an anterior region of interest may have additional clinical relevance. However, since predictions of vertebral strength in compression and in bending are correlated,(43–46) any benefits of limiting measures of vertical tissue fraction to an anterior region of interest are not obvious.

One technical issue related to the loading was the manner in which we implemented the uniform compression. We compressed the vertebrae via thin layers of PMMA applied over each endplate. This ignores any possible influence of the intervertebral disk. While the disk condition has a significant influence on vertebral strength,(28,47) it is unclear whether this influence alters the association between the amount of vertical trabeculae and vertebral strength. Hulme and colleagues reported a similar correlation as reported here between total bone volume fraction and vertebral strength for spine segments of similar age that were compressed biomechanically via a disk.(28) This suggests that the presence of the disk may not appreciably alter the association between bone volume fraction and vertebral strength. Moreover, our finding that the major load paths were parallel columns of vertically oriented bone is consistent with previous work(4,7,9,48) and reflects the overall vertical nature of the loading rather than an artifact of loading via PMMA endcaps. Our previous work suggests that the PMMA endcaps “protect” the vertebral endplates from experiencing high strain.(13) While compressing the vertebra via a disk is expected to place greater loads on the central region of the endplates and on the underlying trabecular bone, the anisotropic structure of the trabecular bone in combination with the vertical nature of the loading suggests that the vertical trabeculae would remain the most structurally important trabeculae and therefore still best explain the variations in vertebral strength. Clearly, additional studies are required to resolve this issue, and more complex loading such as combined compression and forward flexion(41,42) also should be considered.

In summary, our findings show that variation in vertebral strength across individuals is primarily due to variations in the bone volume fraction of vertical trabeculae. This is so because the major load paths in the vertebrae are parallel columns of vertically oriented bone. The vertical tissue fraction—a new indicator of bone quality—is a potentially powerful microarchitectural determinant of vertebral strength.

## References

[1] Mosekilde L, Viidik A, Mosekilde L (1985). Correlation between the compressive strength of iliac and vertebral trabecular bone in normal individuals. Bone..

[2] Mosekilde L, Ebbesen EN, Tornvig L, Thomsen JS (2000). Trabecular bone structure and strength-remodeling and repair. J Musculoskel Neuronal Interact..

[3] Thomsen JS, Ebbesen EN, Mosekilde L (2002). Age-related differences between thinning of horizontal and vertical trabeculae in human lumbar bone as assessed by a new computerized method. Bone..

[4] Liu XS, Sajda P, Saha PK (2008). Complete volumetric decomposition of individual trabecular plates and rods and its morphological correlations with anisotropic elastic moduli in human trabecular bone. J Bone Miner Res..

[5] Liu XS, Bevill G, Keaveny TM, Sajda P, Guo XE (2009). Micromechanical analyses of vertebral trabecular bone based on individual trabeculae segmentation of plates and rods. J Biomech..

[6] Andresen R, Werner HJ, Schober HC (1998). Contribution of the cortical shell of vertebrae to mechanical behaviour of the lumbar vertebrae with implications for predicting fracture risk. Brit J Radiol..

[7] Eswaran SK, Bayraktar HH, Adams MF (2007). The micro-mechanics of cortical shell removal in the human vertebral body. Comput Method Appl Mech Eng..

[8] Eswaran SK, Gupta A, Adams MF, Keaveny TM (2006). Cortical and trabecular load sharing in the human vertebral body. J Bone Miner Res..

[9] Homminga J, Van-Rietbergen B, Lochmüller EM, Weinans H, Eckstein F, Huiskes R (2004). The osteoporotic vertebral structure is well adapted to the loads of daily life, but not to infrequent “error” loads. Bone..

[10] Rockoff SD, Sweet E, Bleustein J (1969). The relative contribution of trabecular and cortical bone to the strength of human lumbar vertebrae. Calcif Tissue Res..

[11] Yoganandan N, Myklebust JB, Cusick JF, Wilson CR, Sances A (1988). Functional biomechanics of the thoracolumbar vertebral cortex. Clin Biomech..

[12] Fields AJ, Eswaran SK, Jekir MG, Keaveny TM (2009). Role of trabecular microarchitecture in whole-verterbal body biomechanical behavior. J Bone Miner Res..

[13] Eswaran SK, Gupta A, Keaveny TM (2007). Locations of bone tissue at high risk of initial failure during compressive loading of the human vertebral body. Bone..

[14] Kopperdahl DL, Pearlman JL, Keaveny TM (2000). Biomechanical consequences of an isolated overload on the human vertebral body. J Orthop Res..

[15] Buckley JM, Loo K, Motherway J (2007). Comparison of quantitative computed tomography-based measures in predicting vertebral compressive strength. Bone..

[16] Eriksson SAV, Isberg BO, Lindgren JU (1989). Prediction of vertebral strength by dual photon-absorptiometry and quantitative computed-tomography. Calcif Tissue Int..

[17] Faulkner KG, Cann CE, Hasegawa BH (1991). Effect of bone distribution on vertebral strength: assessment with patient-specific nonlinear finite element analysis. Radiology..

[18] Lewis G (1997). Properties of acrylic bone cement: state of the art review. J Biomed Mater Res..

[19] Bevill G, Eswaran SK, Farahmand F, Keaveny TM (2009). The influence of boundary conditions and loading mode on high-resolution finite element-computed trabecular tissue properties. Bone..

[20] Adams MF, Bayraktar HH, Keaveny TM, Papadopoulos P (2004).

[21] Eswaran SK, Fields AJ, Nagarathnam P, Keaveny TM (2009). Multi-scale modeling of the human vertebral body: comparison of micro-CT based high-resolution and continuum-level models. Pac Symp Biocomput..

[22] Roux J, Wegrzyn J, Arlot M (2009). Contribution of trabecular and cortical components to biomechanical behavior of human vertebrae: an ex-vivo study. J Bone Miner Res..

[23] Heaney RP (2003). Is the paradigm shifting?. Bone..

[24] Hernandez CJ, Keaveny TM (2006). A biomechanical perspective on bone quality. Bone..

[25] Stauber M, Rapillard L, van Lenthe GH, Zysset P, Müller R (2006). Importance of individual rods and plates in the assessment of bone quality and their contribution to bone stiffness. J Bone Miner Res..

[26] Shi X, Liu XS, Wang X, Guo XE, Niebur GL (2010). Effects of trabecular type and orientation on microdamage susceptibility in trabecular bone. Bone..

[27] Liu XS, Sekhon KK, Zhang XH, Bilezikian JP, Guo XE (2009). Individual trabeculae segmentation based morphological analyses of registered HR-pQCT and µCT images of human tibial bone. Trans Orthop Res Soc..

[28] Hulme PA, Boyd SK, Ferguson SJ (2007). Regional variation in vertebral bone morphology and its contribution to vertebral fracture strength. Bone..

[29] Ito M, Ikeda K, Nishiguchi M (2005). Multi-detector row CT imaging of vertebral microstructure for evaluation of fracture risk. J Bone Miner Res..

[30] Link TM, Bauer J, Kollstedt A (2004). Trabecular bone structure of the distal radius, the calcaneus, and the spine: which site predicts fracture status of the spine best?. Invest Radiol..

[31] Sornay-Rendu E, Boutroy S, Munoz F, Delmas PD (2007). Alterations of cortical and trabecular architecture are associated with fractures in postmenopausal women, partially independent of decreased BMD measured by DXA: the OFELY study. J Bone Miner Res..

[32] Ladinsky GA, Vasilic B, Popescu AM (2008). Trabecular structure quantified with the MRI-based virtual bone biopsy in postmenopausal women contributes to vertebral deformity burden independent of areal vertebral BMD. J Bone Miner Res..

[33] Patel PV, Prevrhal S, Bauer JS (2005). Trabecular bone structure obtained from multislice spiral computed tomography of the calcaneus predicts osteoporotic vertebral deformities. J Comput Assist Tomogr..

[34] Bell GH, Dunbar O, Beck JS, Gibb A (1967). Variations in strength of vertebrae with age and their relation to osteoporosis. Calcif Tissue Res..

[35] Snyder BD, Piazza S, Edwards WT, Hayes WC (1993). Role of trabecular morphology in the etiology of age-related vertebral fractures. Calcif Tissue Int..

[36] Kothari M, Keaveny TM, Lin JC, Newitt DC, Majumdar S (1999). Measurement of intraspecimen variations in vertebral cancellous bone architecture. Bone..

[37] Wegrzyn J, Roux JP, Arlot ME (2010). Role of trabecular microarchitecture and its heterogeneity parameters in the mechanical behavior of ex-vivo human L3 vertebrae. J Bone Miner Res..

[38] Yeh OC, Keaveny TM (1999). Biomechanical effects of intraspecimen variations in trabecular architecture: A three-dimensional finite element study. Bone..

[39] Adams MA, Dolan P (2005). Spine biomechanics. J Biomech..

[40] Eastell R, Cedel SL, Wahner HW, Riggs BL, Melton LJ (1991). Classification of vertebral fractures. J Bone Miner Res..

[41] Adams MA, Pollintine P, Tobias JH, Wakley GK, Dolan P (2006). Intervertebral disc degeneration can predispose to anterior vertebral fractures in the thoracolumbar spine. J Bone Miner Res..

[42] Pollintine P, Dolan P, Tobias JH, Adams MA (2004). Intervertebral disc degeneration can lead to “stress-shielding” of the anterior vertebral body: a cause of osteoporotic vertebral fracture?. Spine..

[43] Graeff C, Chevalier Y, Charlebois M (2009). Improvements in vertebral body strength under teriparatide treatment assessed in vivo by finite element analysis: results from the EUROFORS study. J Bone Miner Res..

[44] Crawford RP, Keaveny TM (2004). Relationship between axial and bending behaviors of the human thoracolumbar vertebra. Spine..

[45] Chevalier Y, Charlebois M, Pahr D (2008). A patient-specific finite element methodology to predict damage accumulation in vertebral bodies under axial compression, sagittal flexion and combined loads. Comput Methods Biomech Biomed Engin..

[46] Buckley JM, Kuo CC, Cheng LC (2009). Relative strength of thoracic vertebrae in axial compression versus flexion. Spine J..

[47] Hansson T, Roos B (1981). The relation between bone-mineral content, experimental compression fractures, and disk degeneration in lumbar vertebrae. Spine..

[48] McDonnell P, Harrison N, Liebschner MA, McHugh PE (2009). Simulation of vertebral trabecular bone loss using voxel finite element analysis. J Biomech..

